# Eco-Friendly Coagulant versus Industrially Used Coagulants: Identification of Their Coagulation Performance, Mechanism and Optimization in Water Treatment Process

**DOI:** 10.3390/ijerph18179164

**Published:** 2021-08-31

**Authors:** Nadiah Khairul Zaman, Rosiah Rohani, Izzati Izni Yusoff, Muhammad Azraei Kamsol, Siti Aishah Basiron, Aina Izzati Abd. Rashid

**Affiliations:** 1 Department of Chemical & Process Engineering, Faculty of Engineering & Built Environment, Universiti Kebangsaan Malaysia, UKM, Bangi 43600, Malaysia; nadiahkz@gmail.com (N.K.Z.); nurul.izzati.izni@gmail.com (I.I.Y.); muhd.azraei24@gmail.com (M.A.K.); aishah@samb.com.my (S.A.B.); 2 Research Centre for Sustainable Process Technology, Faculty of Engineering & Built Environment, Universiti Kebangsaan Malaysia, UKM, Bangi 43600, Malaysia; 3 Makmal Pusat, Syarikat Air Melaka Berhad, Jalan Padang Keladi, Durian Tunggal, Melaka 76100, Malaysia; 4 Department of Civil Engineering, Faculty of Engineering & Built Environment, Universiti Kebangsaan Malaysia, UKM, Bangi 43600, Malaysia; ainaagibs@gmail.com

**Keywords:** aluminum coagulant, chitosan, metal removal, optimization, water treatment plant

## Abstract

The evaluation of complex organic and inorganic coagulant’s performances and their relationships could compromise the surface water treatment process time and its efficiency. In this work, process optimization was investigated by comparing an eco-friendly chitosan with the industrially used coagulants namely aluminum sulfate (alum), polyaluminum chloride (PAC), and aluminum chlorohydrate (ACH) in compliance with national drinking water standards. To treat various water samples from different treatment plants with turbidity and pH ranges from 20–826.3 NTU and 5.21–6.80, respectively, 5–20 mg/L coagulant dosages were varied in the presence of aluminum, ferum, and manganese. Among all, 10 mg/L of the respective ACH and chitosan demonstrated 97% and 99% turbidity removal in addition to the removal of the metals that complies with the referred standard. However, chitosan owes fewer sensitive responses (turbidity and residual metal) with the change in its input factors (dosage and pH), especially in acidic conditions. This finding suggested its beneficial role to be used under the non-critical dosage monitoring. Meanwhile, ACH was found to perform better than chitosan only at pH > 7.4 with half dosage required. In summary, chitosan and ACH could perform equally at a different set of optimum conditions. This optimization study offers precise selections of coagulants for a practical water treatment operation.

## 1. Introduction

Worldwide, the water crisis has become a serious issue as the global population increases and climate changes worsen. By 2030, it has been estimated that 700 million people worldwide could experience an intense water scarcity [1,2]. To overcome the challenges, rapid advancement in water treatment technology is necessary to deliver protected and potable drinking water readily. Drinking water is usually treated via different techniques depending on the quality of the raw water, the degree of contamination, and regulations for public health safeguarding [3]. Among all water treatment technologies, coagulation and flocculation have been an essential part of drinking water and wastewater treatment processes for decades [3,4].

To date, research and development of coagulants have been carried out intensively to improve and establish coagulants with certain properties such as being less hazardous to the environment, having high stability, and being resilient to various process conditions so as to allow maximum coagulation performances [5]. This resulted in immense exploration and comparison between coagulants from both inorganic and organic, which include the traditional industrially used coagulant of aluminum sulfate (alum) that has been the most widely used coagulant, due to its low cost and abundant availability [6]. Other industrially used coagulants are the pre-hydrolyzed inorganic coagulants, namely polyaluminum chloride (PAC) [7], aluminum chlorohydrate (ACH) [7], and ferric chloride (the iron form coagulant) [8,9], which pose inherent advantages such as simplicity in their application and properties, and their ease to control during the manufacturing process [10]. Apart from the inorganic coagulants, organic coagulants such as chitosan have also attracted much attention due to their low cost and environmentally friendly features. Depending on the contaminants treated, the sludge produced from organic coagulant is usually biodegradable and does not create harmful chemical residue effluents [11,12]. The utilization of organic coagulants was also reported to reduce any potential health hazards related to the increase in Al concentration in drinking water that could have possibly occurred from the utilization of inorganic Al-based coagulants. A study reported a potential link between the neurotoxicity sourced from aluminum and its pathogenesis to Alzheimer’s disease [13]. The inorganic coagulants could also cause the generation of a large volume of toxic metal hydroxide sludge, which is also a health hazard due to its carcinogenic property [14]. This has resulted in disposal problems, as well as an increase in an ionic metal concentration of aluminum, specifically in treated water [15]. In comparison to inorganic coagulants, the organic/bio-coagulant of chitosan promises excellent properties and performances with additional values of being more economical, widely available, free of toxins, and biodegradable [16].

Presently, coagulation processes have been intensively studied covering different aspects such as the effect of different types of coagulants [17], the utilization of modified coagulants [18,19], and the effect of process parameters such as pH [20,21] and dosages on water/wastewater treatment [21]. Some of the studies include the comparison between different Al-based inorganic coagulants performances such as between ACH, alum, and PAC [20]. In the study, the effect of pH on the treatment of humic acid (HA) kaolin-synthetic water showed that the coagulants have good performance in acidic conditions [20]. Apart from that, a comparison has also been conducted between inorganic and organic coagulants such as PAC and chitosan, where the dosage and the methods of coagulant addition were investigated [22]. In another study, the application of cationic-grafted starch (St-G) and PAC in low-turbidity micro-polluted surface water has also demonstrated a successful reduction in PAC dosage, when PAC was utilized as the co-coagulant [23]. Even though promising achievements have been reported previously, the application of the improved coagulants to the industries was still quite limited. Industries are still bound to the traditional practice of utilizing common Al-based coagulants such as alum, PAC, and ACH following a standard operation procedure, without properly evaluating the optimum type of coagulants and their optimum parameters that could be utilized. Therefore, a study providing an in-depth insight on application, comparison, and optimization of the different Al-based coagulants commonly utilized in the industries and possible transitioning to natural coagulants to meet drinking water standards is necessary to ensure an economical and sustainable approach could be implemented.

Thus, this work was thoroughly carried out with the aim of identifying the performance of the commonly used Al-based coagulations in the industries and comparing them with an abundantly available, low-cost organic coagulant, such as chitosan for surface water treatment sources with various turbidities, which were obtained from different places in Melaka, one of the states in Malaysia. Focusing on treating surface water is important and still relevant, as presently around 114 million people globally are still dependent on surface water as a drinking water source [24]. To the best of the authors’ knowledge, there are very limited references available that have reported on the performance comparison between chitosan and different types of Al-based coagulants via one-factor-at-a-time (OFAT) and optimization methods. Although the investigated water samples were sourced from Malaysia, the study is believed applicable for surface waters with properties within the turbidity investigated. Commonly, surface water with similar properties as Malaysia’s surface water can be found in countries sharing similar tropical climates with south-western and north-eastern monsoons. Surface water quality has been reported to have been linked with climate change [25]. For example, the amount of rainfall could affect the erosion of soil and transport of fine sediments from land, thus influencing the amount of suspended solids [26], sediment outputs [27], and contaminant metal fluxes [28] in the surface water.

In this study, coagulants of an organic type such as chitosan, and inorganic types such as alum, PAC, and ACH were compared based on their performances in producing the best water quality to meet the standard of national drinking water during the treatment of the raw surface water. The water quality parameters, namely turbidity, metals’ residual concentrations detected in the water sample, were measured before and after the jar test to determine the effectiveness of these coagulants. Real raw water sources that are fed to water treatment plants (WTPs) were obtained from different rivers and dams having turbidity values of 25.6, 130.3, 225, and 826.4 NTU and contain a different concentration of metals. Two different techniques of one-factor-at-a-time (OFAT) and optimization using response surface methodology (RSM) were implemented. Apart from determining the optimum condition to meet the standard of national drinking water, the dominant factors, interaction effects between input factors on the responses, and comparison between organic and inorganic coagulants performance were also investigated. The number of coagulant process parameters investigated included the categorical factor (type of coagulant) and numerical factors (pH and coagulant dosage). The obtained results from the optimization were also compared with the conventional jar test (using OFAT method) to validate the proof-of-concept study. This has not been reported so far and could be highly beneficial for industrial usage. The mechanism of the coagulation/flocculation process upon using organic and inorganic chemicals was also identified and presented.

## 2. Methodology

### 2.1. Samples of Raw Water

Surface water samples from different local river sources in Melaka state, Malaysia, were supplied by the Syarikat Air Melaka Berhad (SAMB), a state government-linked company, which is responsible for water supply services in Melaka, Malaysia. The water samples were mainly taken from three districts in Melaka state to represent the water from each district available in Melaka. The studied samples were taken from Gadek I WTP and Gadek II WTP situated in the Alor Gajah district, from Kesang River situated in the Jasin district and from Gangsa River located in the Central Melaka district. All the samples were characterized immediately after they were received and kept at room temperature (25 °C). The information of the water source and its qualities are listed in Table 1. Initial characteristics of the raw water were identified by using the methods presented in Section 2.2. The national standard of drinking water quality parameters was also included in Table 1.

### 2.2. Coagulant Materials and Preparation Method

#### Coagulants and Preparations

Three different inorganic coagulants,—(1) aluminum sulfate (alum) supplied by See Sen Chemical Bhd., (2) polyaluminum chloride (PAC) supplied by CCM Usaha Kimia (M) Sdn. Bhd., and (3) polyaluminum chlorohydrate (ACH), supplied by Chemkimia Sdn. Bhd.—were used. The organic coagulant chitosan was purchased from Sciencefield Expertise PLT in a solid form. The purchased chitosan had a deacetylation degree of 85%. Coagulants were prepared with concentrations ranging from 5, 10, 15, and 20 mg/L. The concentration of the Al in mg Al/L was based on the Al strength (%) as shown in Table 2. For the aqueous coagulant, the solution was prepared in a 1-L volumetric flask using distilled water. As for the solid coagulant, the magnetic stir was applied to achieve a homogeneous solution. Unlike other coagulants, 10 mL of 0.1 M of hydrochloric acid (HCl) was initially required in the preparation of chitosan solution. Chitosan was left in the HCl solution for 2 h to ensure a complete dissolution, which was then diluted with distilled water to prepare for 1 mg/L chitosan. HCl was chosen instead of acetic acid to ensure that no contaminant of organic matter from the acetic acid to the sample could occur. The purity of the coagulant samples is shown in Table 2.

### 2.3. Jar Test

A jar test was conducted as a batch process and consisted of a multiple flocculator system of the Velps Scientica model VP-F105-A0108, from Italy. Initially, raw water from different sources was mixed homogeneously before it was poured into 1-L beakers of 4 units. Samples were then measured for pH, turbidity, and residual concentrations, after which Fe, Mn, and Al were detected (these could impose risk to health and/or could cause chronic problems to drinking water utilities [30], if presence above the allowable limit in the drinking water). The allowable limits of Fe, Mn, and Al are presented in Table 1. The desired amount of coagulant was then added in the beakers followed by agitation at different mixing times and speeds: rapid mixing of 150 rpm for 1 min, and subsequent slow mixing of 35 rpm for 15 min. The floc formed was allowed to settle for 30 min after terminating the agitation. Finally, the sample was withdrawn from the surface of the supernatant using a micropipette to measure the final turbidity, and the Fe, Mn, and Al concentration. Throughout the test, hydrated lime was used to adjust the pH to 6.5–9.00 as set by the national standard of drinking water quality [29].

### 2.4. Analysis

#### 2.4.1. Turbidity

The measurement of turbidity was conducted using a HACH 2100AN Turbidimeter (HACH Company, Ames, IA, USA) with a 30-mL sample cell. This measurement was based on the light-transmitting properties of the water.

#### 2.4.2. Aluminum, Manganese, and Iron(III)

A syringe was used to withdraw the sample from the surface of the supernatant and the presence for Al, Mn, and Fe, and their concentrations were detected using an Atomic Absorption Spectrometer (AAS) from Perkin Elmer, AAnalyst 800 model.

#### 2.4.3. Response Surface Methodology (RSM)

A two-factor central composite design (CCD) with 4 four replicates at the center point was used in this study to find the best coagulant and optimum conditions for the treatment process of the surface water (Design Expert version 6.0.10). The response surface methodology (RSM) indicated a total of forty-eight (48) runs were needed to complete this design. Raw water from the Gangsa River with a turbidity reading of 225 NTU, pH of 6.68, and metal concentrations of 13.700 mg/L of Al, 3.322 of mg/L Fe, and 0.096 of mg/L Mn was chosen (see Table 1). Different water composition was utilized throughout the study to ensure consistency in the coagulants’ performances. In RSM, the important variables investigated consist of numerical and categorical factors, which represented by coagulant dosage (A), initial pH (B), and type of coagulant (C), respectively. The effects of these variables were investigated against surface water turbidity (NTU) and final concentration of Al, Fe, and Mn (mg/L). The coagulant used for this design was alum, PAC, ACH, and chitosan, and the range and level set for this design was as shown in Table 3. For the pH, hydrated lime was used to increase the pH while HCl was used to reduce the pH.

In the RSM study, all experimental data were analyzed and consistency in experimental and predicted responses were evaluated using a response surface regression procedure to fit a second-degree polynomial equation presented in Equation (1).

(1) $$Y=\beta_{0}+\overset{k}{\underset{i=1}{\sum }}\beta_{i}X_{i\;}+\overset{k}{\underset{i=1}{\sum }}\beta_{ii}X_{i}^{2}+\overset{k-1}{\underset{i=1}{\sum }}\overset{k}{\underset{j=i=1}{\sum }}\beta_{ij}X_{i}X_{j}+\varepsilon \;$$

where *Y* is the predicted response factor, *β* is the model coefficient, *β*_0_ is the intercept term, *β_i_* is the linear coefficient, *β_ii_* is the second order polynomial coefficient, *β_ij_* is the interaction term, and *X_i_*, *X_j_* are the coded independent variables. The analysis of variance (ANOVA) was also carried out to understand the interactions between variables and responses, while the quality of the fit polynomial models was evaluated using coefficient of determination, R^2^. Further analysis on the interactions between factors and variables were also carried out using perturbation plots and 3D response surface diagram. Other statistical analysis namely *F*-test and *p*-value with a 95% confidence level were also utilized to evaluate the statistical significance of the model.

## 3. Results and Discussion

### 3.1. Effect of Different Turbidity Values

**Figure 1:**
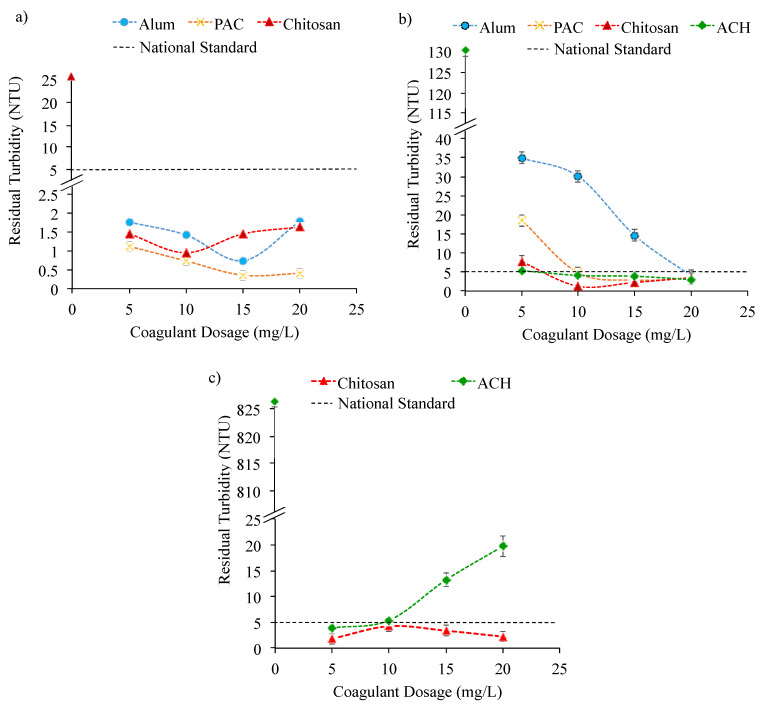
Residual turbidity versus coagulant dosage for different raw water initial turbidity of (**a**) 25.6 NTU (pH 6.63), (**b**) 130.3 NTU (pH 6.80), (**c**) 826.3 NTU (pH 5.21).

**Raw water turbidity of 25.6 NTU:** Figure 1 presents the result of sample water treatment having different turbidity levels of 25.6, 130.3, and 826.3 NTU using both organic and inorganic coagulants. Overall, it was observed that at water turbidity of 25.6 NTU, all coagulants successfully met the standard for national drinking water below 5 NTU, at all dosages investigated from 5 to 20 mg/L. For the low water turbidity, ACH was not tested as the coagulants of lower strength, namely alum and PAC demonstrated excellent performance in treating surface water to the national standard of drinking water. Furthermore, the high cost of ACH has hindered the use of this coagulant for low turbidity water especially when alum and PAC can perform the process well. The organic coagulant of chitosan showed that the highest turbidity removal was achieved at a coagulant dosage of 10 mg/L, while the inorganic coagulants of alum and PAC at 15 mg/L (Figure 1a).

**Figure 2:**
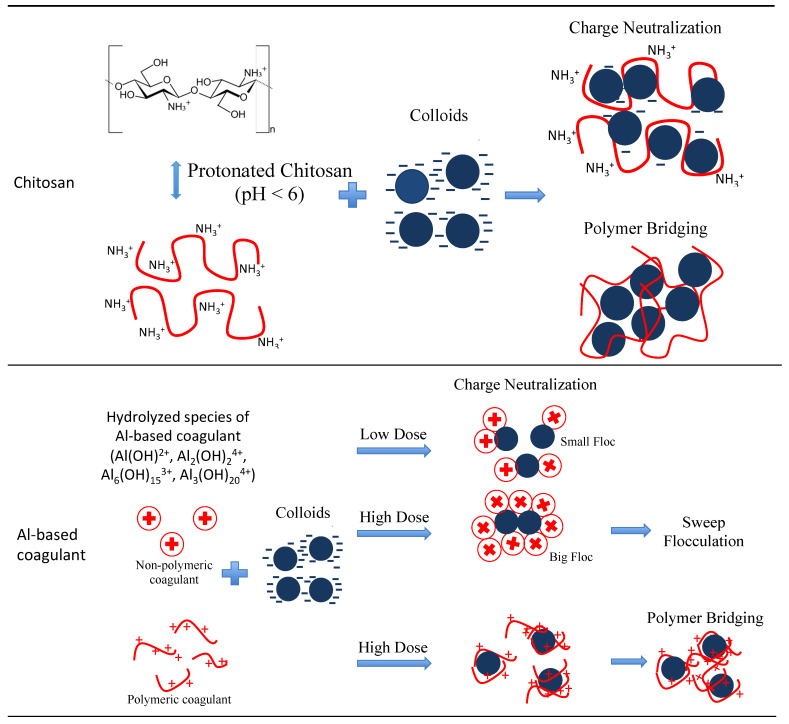
Illustration of coagulation–flocculation mechanisms of chitosan and Al-based coagulants (non-polymeric and polymeric coagulant)**.**

**Raw water turbidity of 130.3 NTU:** At a higher turbidity water of 130.3 NTU, a low coagulant dosage of 5 mg/L did not reduce the water turbidity to the drinking water standard, especially for alum and PAC, which required higher dosages of 20 and 10 mg/L, respectively (see Figure 1b). For PAC, a similar dosage of 15 mg/L as in the low turbidity water (25.6 NTU) was sufficient to achieve the highest turbidity removal. PAC was reported to have shown a better performance than alum. However, when PAC was compared with ACH, ACH demonstrated better results (see Figure 1b). The difference in all the Al-based coagulants’ performances was based on the resultant Al species formed once the coagulants were added to water. Generally, Al^3+^ undergoes rapid hydrolysis reaction with water, and different Al species and Al-hydroxide were formed including monomeric Al species (Al(OH)^2+^, Al(OH)_2_^+^, Al(OH)_4_^−^, (Al_2_(OH)_2_^4+^, Al_3_(OH)_4_^5+^), medium polymerized Al species (Al_13_), and/or high polymerized Al species (Al_30_) [31]. From previous studies, coagulants with a high proportion of stable and high positive electrical charge of Al species such as Al_13_ and Al_30_ could remove contaminants efficiently [15,32]. Al_13_ was reported to have a highly charged cluster with seven positive charges that not only contributed to the effectiveness in the neutralization of contaminants but also acted as nucleate to form floc. Similarly, Al_30_ can effectively absorb negatively charged contaminants and precipitate easily due to its large molecular weight [31]. This phenomenon was found in accordance with the results obtained in this study, in which the coagulants’ performance can be sequenced as alum < PAC < ACH. From Figure 1b, the drinking water standard of 5 NTU could be met at only 5 mg/L dosages for ACH compared to PAC and alum, which did not manage to comply with the standard at low coagulant dosage. The observation is also supported in previous studies [31,33]. Detailed discussion on the difference in the Al coagulation mechanisms is described and illustrated in Section 3.2.1 and Figure 2, respectively. Apart from the inorganic coagulants investigated, the tested organic coagulant of chitosan also showed a good performance in reducing water turbidity to below 5 NTU at a low coagulant dosage. From Figure 1a,b, the chitosan showed that the highest turbidity removal can be achieved at 10 mg/L. The dosage was found consistent for turbidity water at 25.6 and 130.3 NTU. The less coagulant dosage requirement for chitosan compared to Al-based coagulants to achieve the drinking water standard is believed to be due to chitosan possessing high-charge density. Thus, a small amount of the dosage is already sufficient to destabilize particles. Similar results were also observed in the previous study [34,35,36], in which chitosan showed better effectiveness in a coagulation process at a lower dosage compared to the Al-based coagulants, with the additional advantages of being non-hazardous and more environmentally friendly [37,38].

Raw water turbidity of 826.3 NTU: As the chitosan and ACH demonstrated good performances in achieving residual turbidity as required for the drinking water standard, these coagulants were further tested at higher raw water turbidity of 826.3 NTU. From Figure 1c, even at high initial 826.3 NTU water’ turbidity, chitosan could successfully maintain the residual turbidity of below 5 NTU at dosage below 10 mg/L. Such a phenomenon may be explained based on the different pH values of the water samples taken from the WTP, because every turbidity recorded a different pH value. The highest turbidity of 826.3 NTU has a pH of 5.21 (see Table 1). Therefore, the good performance of chitosan even at high water turbidity of 826.3 NTU, may be contributed from the high acidity of the water sample. In an acidic condition (at pH < 6), chitosan usually demonstrates high zeta potential of more than 20 mV [39]. At this condition, the amine (NH_2_) functional groups present in chitosan are highly positively charged and they can effectively bind and bridge the negatively charged contaminants and colloidal materials. This could provide electrical neutrality at a low dosage via adsorption, charge neutralization, inter-particle bridging, and hydrophobic flocculation [37,40]. The presence of high number of protons in the solution at low pH conditions could protonate more amine groups of the chitosan molecule, thus, create more sites for adsorption of contaminants/metals. Moreover, flocs produced by chitosan coagulation were also reported to form and grow rapidly to a large size, thus easing the sedimentation process [38]. Therefore, such a phenomenon explained the high turbidity removal of chitosan even with a water sample with high turbidity of 826.3 NTU.

Chitosan also shows good performance regardless of the coagulant dosage, which is believed to be due to the water sample’s high acidity of pH 5.21 that favors the chitosan coagulation process. The mechanism illustration of a coagulation–flocculation process using chitosan is also presented in Figure 2, which is further discussed in the later section of Section 3.2.1. In contrast, as for ACH, at an increasing dosage, the turbidity removal performance of the coagulant reduced, which indicated that the coagulant performance worsened. In a low pH (acidic) sample, ACH was found to work best at a low coagulant dosage. At increasing coagulant dosages, charge reversal and possible destabilization of colloid particles may have occurred because of the possible occurrence of a significant reduction in pH of a sample when a high coagulant dosage was utilized [41]. In addition, the pH could affect the stabilization of a suspension. A similar result was also observed in the previous studies [41,42]. Meanwhile for the chitosan, charge reversal was not observed at an increasing dosage because at pH 5.21, only partial protonation may have occurred; thus, sufficient charge colloid and fine particles in the sample were available for attachment and polymer aggregation. As reported in other studies, chitosan could reach approximately 90% amine protonation at pH 4, but reduces to 50% as the pH increases to 6 [43,44].

#### Coagulation–Flocculation Mechanism of Chitosan and Al-Based Coagulant

From the results presented in the previous section, both inorganic and organic coagulants could meet the requirements of the national drinking water standard upon applying them at different coagulant dosages. However, the organic coagulant of chitosan demonstrated a low dosage requirement of only 10 mg/L compared to the Al-based coagulants investigated such as alum, PAC, and ACH, which were found to be effective at a higher dosage at the condition of the raw water investigated. The differences in the coagulants’ performances can be described based on the coagulation–flocculation mechanisms, which are mainly influenced by the coagulant’s molecular composition and properties. The illustration of the mechanisms is presented in Figure 2. Chitosan is a linear polysaccharide that consists of monomers of 2-amino-2-deoxy-D-glucose connected by α−1 → 4-linkages. The amino groups (-NH_2_) at the C-2 position can be protonated in acidic conditions resulting in a cationic property. Therefore, as chitosan is highly packed with positive charges, the coagulation mechanism worked by neutralizing the charge contaminants by stages, which consequently resulted in patches of neutralized micro-flocs. During the neutralization process, the repulsive interactions of the similarly-charged contaminants were reduced leading to destabilization of the contaminants [45]. This mechanism is followed by bridging the micro-flocs together through electrostatic binding with different loci available along the linear chain of the polysaccharide chitosan forming macro-flocs, which were then aggregated and settled [36]. The ability of chitosan to undergo charge neutralization and the inter-particle bridging mechanisms with the contaminants contributed to the low requirement dosage of chitosan compared to the Al-based coagulant for effective coagulation process. However, it is important to note that at an increasing pH of a solution, different coagulation mechanisms of metal hydroxide may apply for chitosan. According to Blockx et al., (2018), chitosan started to precipitate at pH > 7.5 [39]. A similar study was also reported by Demir et al., (2020) on the flocculation of microalgae in chitosan, where effective flocculation was observed at pH < 6.5 based on the electrostatic interactions between chitosan and the negative cell surface of microalgae. On the other hand, a different mechanism was found upon increasing the pH with metal hydroxides whereby precipitation of chitosan occurred instead of flocculation [46].

In contrast, for the Al-based coagulants, the coagulation–flocculation process mainly evolved around charge neutralization mechanism. The contaminants need to be sufficiently large to form nuclei, especially for coagulants with a high fraction of monomeric Al species such as alum [47], as discussed in the previous Section 3.2. If an insufficient size of flocs was formed at a low dosage of the Al coagulant, no inter-particle bridging could happen, and thus no precipitation could occur through sweep-flocculation. This phenomenon usually occurred for coagulants with high monomeric Al species as the monomeric species is the most unstable species. As the dosing increases, the monomeric Al species could be transformed into more stable polymeric and colloidal species of Al_13_ and Al_30_ facilitating the growth of flocs via bridging [31]. Simultaneously, the complexation of contaminants with coagulant also occurred, reducing the negative charge of the contaminant. Consequently, the collision frequency and efficiency were favored, thus contributing to the growth of flocs [31]. The illustration of the mechanism can be seen in Figure 2. This is also true for polymeric coagulant, PAC, which undergoes a bridging mechanism. A sufficient amount of coagulant is needed for bridging to occur between the neutralized colloids [38] as illustrated in Figure 2. This phenomenon clearly reflects the result presented in Figure 1b, where at a water turbidity of 130.3 NTU, the increase in alum and PAC dosages significantly reduced the water turbidity.

### 3.2. Effect of Metals Removal in Treated Water

#### 3.2.1. Raw Water Turbidity of 25.6 NTU

Apart from the turbidity removal discussed in the previous section, the coagulants’ performances in removing residual metal concentrations such as Al, Mn, and Fe in the treated water was also investigated to ensure that their limit complies with the national standard of drinking water at Al, 0.20 mg/L, Mn, 0.10 mg/L, and Fe, 0.30 mg/L [29]. Exceeding the allowable limits could impose hazards to human health, the environment, and affect the properties of the drinking water.

**Figure 3:**
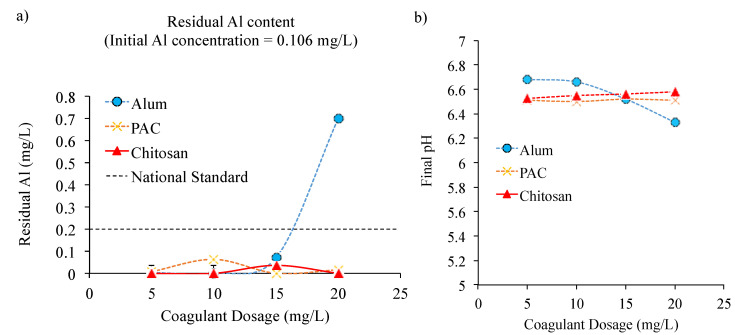
(**a**) Concentration of residual Al, Fe, and Mn in low water turbidity water using alum, PAC, and chitosan at dosages from 5 to 20 mg/L, and (**b**) the final pH after coagulation process using alum, PAC, and chitosan.

In the low water turbidity of 25.6 NTU, initial metal concentrations of Al, Mn, and Fe were found to be at 0.106, 0.062, and 0.142 mg/L, which were already below the allowable limit of the national standard of drinking water (see Figure 3). Thus, for the 25.6 NTU water sample, only the effect of the Al-based coagulants’ dosage on the residual Al concentration was investigated. From the results, after treating the water with Al-based coagulants, namely alum, PAC, and chitosan, there was no increase in residual Al concentrations; instead, the Al concentration was further reduced. Such a phenomenon indicated that the coagulation process was found effective at various coagulation dosages and process conditions of pH 6.63, in which flocs were readily formed and removed [33]. However, this phenomenon was only true for an alum coagulant concentration presence of up to 15 mg/L, beyond which, the residual Al in the treated water increased more than its initial value of 0.106 mg/L. At a high coagulant dosage, the hydrolysis of the alum reduced the final pH of the treated sample as shown in Figure 3b. This could possibly lead to the formation of some soluble Al species, thus increasing the residual Al concentration in the treated water [48]. A similar result was also observed in the previous reported studies [33,49].

#### 3.2.2. Raw Water Turbidity of 130.30 NTU

**Figure 4:**
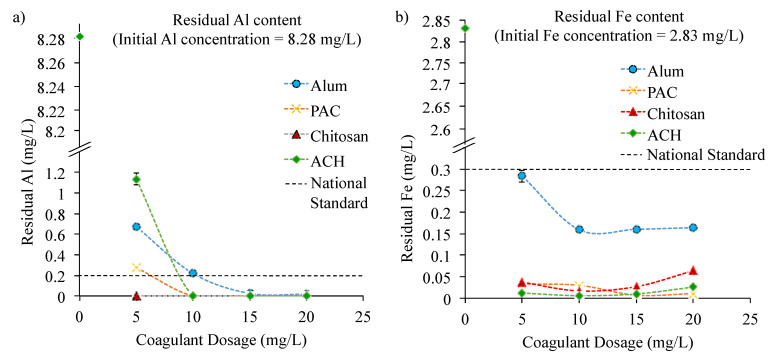
Concentration of (**a**) Al and (**b**) Fe in the treated water (initial water turbidity: 130.30/NTU, pH: 6.80; initial metal concentration: Al, 8.28 mg/L; Mn, 0.09 mg/L; Fe, 2.83 mg/L; national standard of drinking water content; Al = 0.20 mg/L, Mn = 0.10 mg/L and Fe = 0.30 mg/L).

Meanwhile, for samples with initial water turbidity of 130.30 NTU, the concentration of the residual Al and Fe are presented in Figure 4. The residual concentration of Mn was not discussed, as its concentration was already sufficiently low, at about 0.09 mg/L (below the standard of 0.10 mg/L). For this water sample, the effect of using four different coagulants for the removal of metal concentrations was investigated namely alum, PAC, chitosan, and ACH. Overall, Figure 4 shows that chitosan successfully reduced the concentration of Al to the standard of drinking water at all investigated dosages from 5 to 20 mg/L.

In contrast, for the Al-based coagulants (alum, PAC, and ACH), a low dosage of 5 mg/L was found to be insufficient to reduce the Al concentration in the raw water sample to the allowable value of below 0.2 mg/L. It was observed that a higher dosage of above 10 mg/L was required, except for the water treated with alum. The residual Al recorded in the alum-treated water sample was 0.22 mg/L, slightly higher than the set standard of 0.20 mg/L. This result was found to be consistent with the result obtained in the previous Section 3.1 on the turbidity removal and the coagulation–flocculation mechanism presented in Figure 2. The higher effectiveness of ACH and PAC compared to alum is because they both possessed higher basicity than alum (0% basicity), with 0% basicity in alum indicating that alum has no –OH group in its structure that could increase its charge density for an effective contaminant removal [7]. Moreover, ACH and PAC also possess a high fraction of medium and high polymerized Al species of Al_13_ and Al_30_ that have low solubility and a large surface area to allow easy adsorption and co-precipitation of the contaminants [50]. This has resulted in the effective removal of Al (see Figure 4a). Kimura et al., (2013) reported that coagulant with the highest basicity demonstrated the lowest residual Al concentration in the final sample [50]. Generally, the increase in basicity can be correlated with the increase in the percentage of high polymerized Al species, Al_30_ in a coagulant [50]. These findings were also in accordance with the properties of the coagulants utilized in this study as presented in Table 1. Meanwhile, the alum demonstrated the effective removal of Al of up to 99% when the coagulant dosage was increased to more than 10 mg/L (see Figure 4a).

In removing Fe in the water samples to the allowable limit (<0.30 mg/L), it was found that all coagulants including chitosan, ACH, and PAC have performed the process well (see Figure 4b). In Fe removal using chitosan, the mechanism of retention involved a complex phenomenon of lump formation on the structure of chitosan with protonated groups of –OH_2_^+^ and –NH_3_^+^. The strong coordination of Fe was formed with chitosan functional groups through surface adsorption [51]. A similar trend in Al removal was observed for Fe removal, which alum coagulant demonstrated as the least effective coagulant. Figure 4b shows that at 5 mg/L, the alum removal percentage was the lowest, at 90% (0.28 mg/L), compared to the other coagulants with a removal percentage of more than 98%. Moreover, at an increasing alum dosage of more than 5 mg/L, its effectiveness in Fe removal could only reach up to 95%. This is probably because alum has zero basicity properties and its lower fraction of the Al_13_ reduces alum’s effective neutralization process of contaminants. For the other coagulants such as PAC, the highest removal of Fe is obtained at 15 mg/L, while the ACH and chitosan are at 10 mg/L.

#### 3.2.3. Raw Water Turbidity of 826.3 NTU

**Figure 5:**
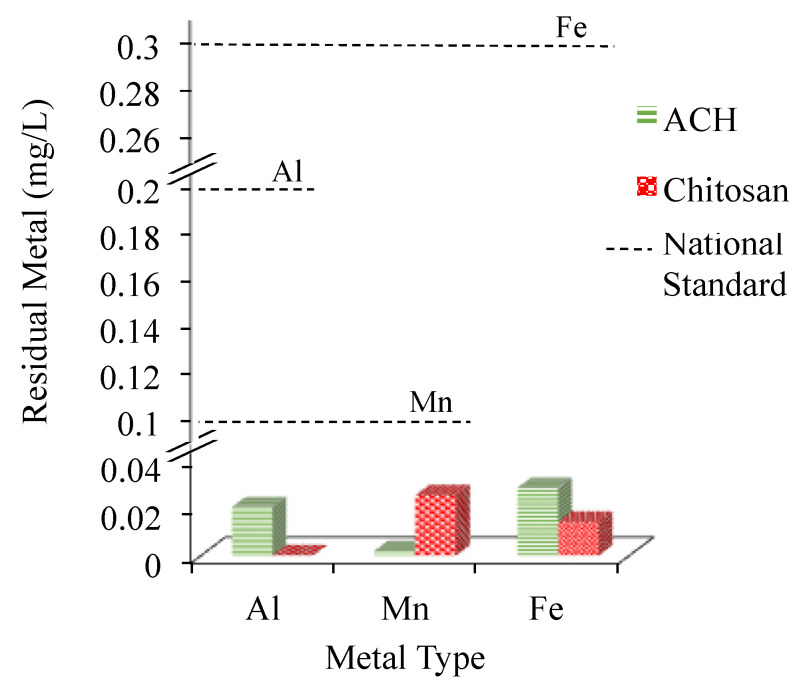
Residual concentration of Al, Mn, and Fe in treated water using 10 mg/L coagulant dosage of ACH and chitosan (initial water turbidity: 826.30 NTU, pH: 5.21; initial metal concentration: Al, 75.14 mg/L; Mn, 0.10 mg/L; Fe, 7.70 mg/L; national standard of drinking water content; Al = 0.20 mg/L, Mn = 0.10 mg/L, and Fe = 0.30 mg/L).

For the treatment of the water sample with the highest water turbidity of 826.3 NTU, two of the best coagulants, namely chitosan and ACH at 10 mg/L were utilized, and dosage was determined based on the highest removal of metals obtained in the previous Section 3.2.2. As seen in Figure 5, at 10 mg/L, both coagulants successfully reduced the concentration of all metals to the concentration permitted by the Ministry of Health, Malaysia (MOH), consistent with the results obtained for turbidity removal of the water with low turbidity of 130.30 NTU. All metals were removed greater than 99%. Such result is possibly obtained because the ACH, a pre-hydrolyzed Al-based coagulant, and chitosan worked well in a wide pH range between 5.0 and 7.5 and at low coagulant dosage [52]. Therefore, despite the presence of higher metals concentration in the water samples with a turbidity of 826.3 NTU compared to other water samples with lower turbidity value, the pH of the water at 5.21 was still within the effective pH for ACH and chitosan coagulant efficacy. Previous studies have demonstrated that pH has significant effects on the coagulation process, in which pH influenced the hydrolysis, polymerization, and the resultant species of coagulants [13,53]. As for the Al-based coagulant, the hydrolysis of the coagulant was linked to the reduction coordination number of the Al. Therefore, different hydroxyl species of Al could be produced, which consequently affected the performance of the coagulant and the concentration of residual Al in the coagulation system [53].

Meanwhile, an effective reduction of the metal content in the water sample to the acceptable limit was successfully achieved upon using chitosan, regardless of their initial metal concentrations (see Figure 5). This result is found consistent with the results obtained in the turbidity removal using chitosan as discussed in Section 3.1 and also in the previous study reported utilizing chitosan for Fe and Mn removal [54]. In their study, Mn and Fe removal using chitosan was found to be dependent on the dosage of coagulant and pH, where the metal removal increased with the increase in sample pH from 2.5 to 7 [54]. This may be attributed to the presence of a large number of protonated primary amino onto chitosan polymer chains that allow effective adsorption of metal ions. However, according to Babel et al., (2003), the protonation of primary amino groups is not the only factor contributing to this removal. The excellent metal-binding capacity of chitosan is also influenced by the flexible structure of polymer chains which is dependent on the pH of the solution [55]. In the other study reported by Guibal et al., (2004), the amino sites were found as the main reactive groups for metal ions adsorption, while hydroxyl groups may contribute to sorption only [56]. Even though both ACH and chitosan showed good performances, the utilization of chitosan as a coagulant is a step forward to eliminate the possibility of adding the Al compound that is abundantly present in the Al-based coagulant to the treated water.

### 3.3. Jar Test Optimisation

The response surface method (RSM) was also utilized in this study to further investigate the effect of factors including coagulant dosage, initial pH of water samples, and type of coagulants on the responses, which are the residual turbidity and metal concentration (Al, Mn, Fe). These include understanding interaction trends and behavior of the factors on the responses. Ranges and variables utilized in this section are included in Table 3. A complete set of the experimental design with two-factor CCD (four replicates) is presented in Supplementary Table S1, while the final model predictions presented based on the categorical factors are presented in Table S2. The prior table presents the actual and predicted responses from the interactions of factors, while the latter describes the consistencies between the experimental and predicted responses.

#### 3.3.1. ANOVA for Response Parameter

Table 4 displays the results of the analysis of variance (ANOVA) of the developed models. From the ANOVA, it can be seen that all responses of turbidity, Fe, and Al, except Mn, have significant relationships with the variable dosage, the initial pH, and the type of coagulant. This was determined from the *F*-values and *p*-values generated from equations presented in Table S2. *F*-values obtained from the developed models were found to be greater than the critical *F*-value (2.004 at 95% significance) that was obtained from the standard distribution table with a degree of freedom equal to 14 and 33. Turbidity, Fe, and Al responses showed large *F*-values of 739.70, 14.12, and 63.34, respectively, which confirmed the sufficiency of the developed models. Ghafari et al., (2009) stated that a value for adequate precision of higher than four (4.0) indicated an adequate and desirable signal [57], which supported the findings. Similar results were also demonstrated by the *p*-values *(p*-values < 0.05), indicating that the model equations for turbidity, Fe, and Al were statistically significant at the 95% confidence level. However, for Mn, the model equation was not statistically significant. This was demonstrated by the low *F*-values of 1.18 and *p*-values > 0.05 (see Table 4). Such values were observed probably due to the initial Mn concentration in the water sample was already low, around 0.096 mg/L, lower than the requirement of the drinking water standard of 0.1 mg/L. In general, coagulation of low pollutant loading, such as Mn in this study, is difficult as only a few flocs are available to form larger size flocs or colloids, but some may be of low density. Thus, good removal performance is hard to achieve since difficulty might occur in inducing collision between the colloids [58,59]. Such phenomenon may have resulted in the undesirable model equation.

Apart from the *F*-value and *p*-value, the lack-of-fit of the model regressions signifying the difference between experimental results and model predictions were also evaluated. From Table 4, the critical *F*-value for the Lack-of-Fit determined from the standard distribution table with degrees of freedom equal to 21 and 12 were found to be at 2.54 (95% significance), higher than the *F*-values of the lack-of-fit for the quadratic model regressions. This indicated that the models are fit to be used to predict the responses in this study, which include turbidity and metal residual concentrations of Fe and Al.

The validity of the model equations was also further analyzed using the R^2^ values. The results showed that the model predictions were in good agreement with the experimental values. The regression models could provide a reasonable fit for models containing a different number of independent variables and at making predictions, except for Mn (R^2^ = 0.33). Table 5 shows that the correlation coefficient, R^2^ value for turbidity, and residual concentration of Fe and Al were at 0.99, 0.86, and 0.96 respectively, which were all in an acceptable range. The adequate precision values obtained were also > 4, suggesting reliable experimental data were collected in the study. The R^2^ results further supported the *F*-value and *p*-value obtained, which were discussed in the previous paragraph.

#### 3.3.2. Effects of Interaction Factors on the Responses

Interaction factors for the two best coagulants, ACH, and chitosan on the responses of turbidity and Al and Fe concentrations were also investigated using perturbation plots and 3D-response surface diagrams. However, the influence of factors on Mn concentration was not evaluated as Mn model regression was found to be insignificant, as discussed in Section 3.3.1.

**Figure 6:**
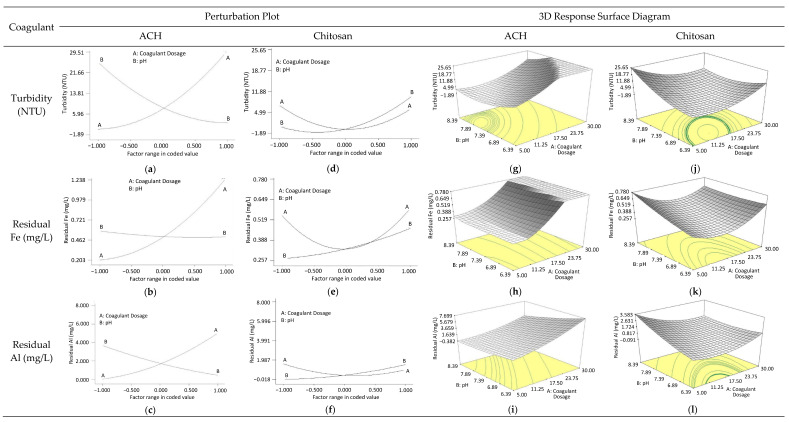
Perturbation plot (**a**–**f**), and 3-dimensional response surfaces diagram with contour plot (**g**–**l**); residual Fe, and residual Al concentration responses toward changes in coagulant dosage and pH using ACH and chitosan as coagulants.

Perturbation Plots: Figure 6 shows the perturbation plots comparing the responses’ effect (turbidity and residual metal concentration) from the change of factors (coagulant dosage and pH) over the range specified when chitosan and ACH were utilized as coagulants. A mid-point has been set as a reference point for all factors to investigate the sensitivity of the responses towards the input factors based on the steepness of the perturbation slope. Overall, Figure 6 demonstrates that the coagulant dosage plays an almost equal role as pH in determining the turbidity and residual metal removals from the water samples. This is indicated by an almost similar steepness obtained in the slopes of line A and line B, especially for the turbidity and Al responses. However, coagulant dosage and pH exerted opposite effects on turbidity and metal removal. The turbidity and metal removal were reduced as the coagulant dosage increased but the opposite effect was seen with the increase in pH. The reduction in turbidity and metal removal with the increased dosage could possibly be due to the occurrence of coagulant charge reversal at high dosage and particle restabilizing [32]. Meanwhile, for the effect of pH, Yang et al., (2010) previously reported that at low pH in a range of below 5 and up to 8 (weak alkalinity), effective turbidity, and metal removal could be observed. This happened because at this condition, the positive hydrolyzates such as Al(OH)^2+,^ Al(OH)_2_^+^, Al_2_(OH)_2_^4+^, Al_3_(OH)_4_^5+^, high polymeric positive hydrolyzates, and Al(OH)_3_ are present_._ Therefore, the negative charged contaminants are easier to be neutralized by the positive hydrolyzates. The high polymeric hydrolyzates also have low solubility and large surface area, which could allow easy adsorption and co-precipitation of the contaminants. In their study, destabilization occurred at a higher pH because of the presence of a high fraction of soluble Al(OH)_4_^−^ [20]. However, in relation to this study, the opposite trend was observed, both turbidity and metal removal was seen to occur effectively at a higher pH condition (see Figure 6a–c). This discrepancy may be explained due to the presence of a different distribution of Al speciation in the coagulants, which the ACH coagulant used in this study has a higher distribution of high polymerized Al species of Al_30_ compared to PAC coagulant utilized in [20]. A similar discussion can be found in the previous Section 3.2.2. With a high distribution of medium and high-polymerized Al species in the ACH coagulant compared to PAC, the ACH coagulant could work efficiently at a high pH value. This finding explained the observed increasing turbidity and metal removal with increasing pH as shown in Figure 6a–c. These findings are the same as reported in previous studies [50,60]. These studies demonstrated that coagulants owing high basicity with more stable preformed Al species were found to work effectively at a higher pH. However, it is important to note that, to achieve the drinking water standard, a balance between the coagulant dosage and the pH value is required.

Meanwhile, for the residual Fe, the response shows a higher sensitivity towards changes in the coagulant dosage compared to pH, especially for the ACH coagulant. This can be clearly seen in Figure 6b. Line A represents that the coagulant dosage displayed a steepness of several magnitudes higher than line B, which almost plateaued throughout the range investigated. The differences in the responses obtained with the change in the input factors were possible because of the different coagulation mechanisms that occurred at different process conditions. This was discussed in the previous Section 3.1 and Section 3.2.2.

Nevertheless, when perturbation plots of chitosan were compared to ACH, they have demonstrated lower response sensitivity towards the input factors (pH and coagulant dosage). This is evaluated based on the steepness of the lines presented in Figure 6d–f, which were less steep than the ACH (see Figure 6a–c). This difference in sensitivity of the responses to the changes in input factors may be due to the chitosan coagulation mechanism that did not depend on the hydrolysis of the coagulant, unlike the Al-based coagulants, which was described in Section 3.2.1. On the contrary to ACH, the responses for pH when chitosan coagulant was utilized acted the other way round. At an initial pH of below 7.39, an increase in the coagulant dosage improved the performance of chitosan, wherein the turbidity and residual metals removal increased with dosage. However, at initial pH of above 7.39, the trend was vice versa. The increase in chitosan dosage increased the residual turbidity of the samples. This can be explained by the precipitation of chitosan out of the solution as the pH reached > 7.5 [39]. According to Blockx et al., (2018), chitosan started to precipitate due to the partial deprotonation of amine groups, where the chitosan lost its positive charge. This contributed to the decreased in the chitosan solubility that consequently resulted in the precipitation of the chitosan [39]. Figure 6d–f shows that the performance of chitosan is highly dependent on the acidity of the water sample. As discussed in Section 3.2.1, the chitosan coagulation effectiveness is dependent on the protonation of the available amine functional groups in the chitosan molecular structure. Apart from that, as seen in Figure 6d–f, the increased in coagulant dosage in acidic water sample condition also significantly reduced the turbidity of water sample below 5 NTU and the residual metals to the allowable limit, which supported the results obtained in the OFAT method. The technical and quantitative analysis from the comparison of the perturbation plots between ACH and chitosan provided clear evidence on how the responses reacted to the changes in the input factors when different coagulants were utilized.

3D Surface Response Diagram: Analysis of the optimization process was further carried out using the 3D response surface diagram presented in Figure 6g–l. Overall, the 3D diagrams were found to be in accordance with the perturbation plots, in which the interaction effects of input factors on the responses can be clearly seen. Figure 6g–i shows that at low ACH dosage < 11 mg/L, the pH of the water sample has a minimum effect on the performance. However, as the dosage increased, the effect of pH on the responses became significant, especially at a low pH value. Such a phenomenon was probably due to, at a higher dosage of coagulant, more hydrogen ions being produced from the hydrolysis of metal ions to form an aluminum hydroxide floc. The large number of hydrogen ions present in the water sample could react with the alkalinity of the water, rapidly decreasing the pH, and thus could interrupt the removal process of turbidity and metals [52]. Generally, if the water sample has a low initial pH value, the alkalinity of the water may not be sufficient to react with the excess hydrogen ions from the high dosage of coagulants utilized; thus, the efficiency of the coagulation process could be significantly interrupted in reducing the turbidity level of the water sample (see Figure 6g). On the other hand, by having a high initial pH of the water sample, the high alkalinity of water could sufficiently react with the increasing amount of hydrogen ions to maintain the efficiency of the coagulation process. This can be clearly seen in Figure 6g, where turbidity removal was least affected by the changes in the coagulant dosage at a high pH value. The ACH coagulant was found to work optimally at a pH between 7.30–7.80 and dosage between 6.60–11.10 mg/L. This result is also consistent with the ACH result obtained in OFAT method, in which the highest turbidity removal could be obtained at 10 mg/L, within the optimum coagulant dosage determined by the RSM (6–11.10 mg/L).

Meanwhile, for chitosan, the optimization study showed that chitosan worked optimally in acidic conditions with a pH around 6.41–6.57, which was consistent with the perturbation plot and results obtained using the OFAT method. However, the optimum dosage of chitosan needed to achieve the drinking water standard determined from optimization (18.00–19.70 mg/L) was higher than the result obtained in the OFAT method (10 mg/L). Such results awee obtained due to the sufficiently lower pH of the water sample used in the OFAT (at pH of 5.21) compared to the studied pH utilized in the optimization of between 6.39 and 6.89. Previous studies reported that the protonation of NH_2_ in chitosan molecular structure could reach up to 90% at pH 4 and regularly decreased to about 50% at pH 6 [43,44]. The reduction in NH_2_ protonation indicated that the positive charge on chitosan also decreased; thus, only fewer particles could be destabilized via the charge neutralization mechanism initiated by the chitosan for the turbidity and metal removal [43,44]. This explained the higher dosage of chitosan needed at a high initial pH (6.39–6.89) used in the optimization study compared to the low pH (5.21–6.80) of the water sample used in the OFAT study. Moreover, when chitosan was utilized as a coagulant in comparison to ACH, the pH played a significant role in achieving the national standard of drinking water.

#### 3.3.3. Model Validation and Comparison with the OFAT Method

In order to validate the model and optimization process, an experimental run was carried out at the predicted optimum inputs generated by CCD, RSM. All the responses, namely turbidity, Mn, Fe, and Al, were set to achieve below the national standard of drinking water at 5 NTU (turbidity), 0.1 (Mn), 0.2 (Al), and 0.3 mg/L (Fe). Meanwhile, the input factors—(i) coagulant dosage, (ii) initial pH, and (iii) type of coagulants—were all set within the studied parameters as presented in Table 3. From the combination of categorical factor and numerical variables designed by CCD for the model validation, the coagulants of ACH and chitosan demonstrated a high desirability (D) of D = 1. The conducted experimental work performed at the optimal input conditions using ACH (categorical factor) at pH 7.40 and dosage at 7.09 mg/L (numerical variable) was found to successfully reduce the turbidity and metal concentration of the treated water to the permissible limit. The turbidity removal was achieved up to 95%, while Mn and Al recorded more than 99% removal, and finally Fe showed more than 90% removal. Similar results were also obtained when chitosan was utilized at the optimum pH of 6.41 and dosage of 18.63 mg/L. The water sample was successfully treated to the national standard of drinking water.

## 4. Conclusions

In conclusion, the performance comparison between chitosan and the commonly used inorganic coagulants in treating some actual surface water samples from different WTPs to meet the national standard of drinking water were successfully investigated. Even though the application of chitosan, the non-toxic coagulant, and the Al-based coagulants, namely alum, PAC, and ACH, is well known in the water treatment process, a comprehensive comparison study between these coagulants was still limited. Overall, in this study, the one-factor-at-time (OFAT) method and the optimization results showed that the coagulants’ physicochemical properties have a significant influence on the performance of the coagulants. This is reflected in the minimum requirement of the ACH and chitosan dosage (10 mg/L) in the removal of the turbidity and metal ions to achieve the standard of drinking water. ACH outperformed other Al-based coagulants due to its higher fraction of respective medium and high polymerized Al species of A_l3_ and Al_30_, which allow easy adsorption and co-precipitation of contaminants. Meanwhile, for chitosan, the good performance in turbidity removal was due to its high positively charged structure. However, chitosan was found to work well at a low dosage in acidic conditions, while ACH worked well in basic conditions. As the pH of the water sample increases, a higher dosage of chitosan is required to meet the national standard of drinking water. This explained the high optimum chitosan dosage needed of 18.58 ± 0.45 mg/L in the optimization study (pH: 6.39 and 6.89), compared to the OFAT method (pH: 5.21 and 6.8). As for ACH, the inorganic coagulant demonstrated a large range of working pH from 5.21 to 8.39 for a minimum ACH dosage of 10 mg/L. Moreover, the study demonstrated that for ACH, critical operational measures might be applied in contrast to chitosan. This is due to the perturbation plot and the 3D response surface diagram from the RSM optimization study, which demonstrated that all responses investigated were more sensitive to the input parameters when ACH coagulant was utilized. From this study, the application of RSM using CCD for jar test optimization showed that different coagulants have different optimum conditions to meet the national standard of drinking water, where the water sample conditions, such as pH, are crucial in determining the type of coagulant utilized and its dosage. The use of alternative coagulants, namely chitosan, could give comparable performance as the inorganic ACH coagulant but at a different optimum condition. It can be also concluded that the RSM could assist in providing highly precise conditions of the coagulation parameters for water treatment and in-depth insight on the influence of the factors and their interaction on the responses.

## Figures and Tables

**Table 1:** Water samples and their characteristics.

Water Sample	pH	Turbidity (NTU)	Aluminum (mg/L)	Iron (mg/L)	Manganese (mg/L)
Gadek II WTP	6.63	25.6	0.11	0.14	0.06
Kesang River	6.80	130.3	8.28	2.83	0.09
Gadek I WTP	5.21	826.3	75.10	7.70	0.10
Gangsa River	6.68	225.0	13.70	3.32	0.096
National standard of drinking water quality [29]	6.5–9.00	5.0	0.20	0.30	0.10

**Table 2:** Coagulants’ properties and grades.

Coagulant	Specific Gravity	Purity (%)	Strength (%)			Chloride (%)	Grade	Cost (Ringgit Malaysia/Metric Ton)
			Al_2_O_3_	Al	Basicity			
Aluminum sulfate (alum)	1.31	26–28	8	4	0	-	Industry	330 1900 *
Polyaluminum chloride (PAC)	1.22	22	10	5.6	50	10–10.5 *	Industry	740 10,590 *
Aluminum chlorohydrate (ACH)	1.35	30–60	23–24	12	82	7.9–8.4	Industry	2350 11,860 *
Chitosan 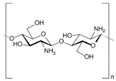	-	100	-	-	-	-	Laboratory, 85% DD, 900 kDa	5630

* Typical values and costs referred from [7].

**Table 3:** Range and level for RSM.

Categorial Factor	Numerical Variable	Range and Level		
		−1	0	1
**C, Coagulant**	A, dosage (mg/L)	5.00	17.50	30.00
	B, initial pH	6.39	7.39	8.39

**Table 4:** ANOVA results for the four responses including turbidity and residual concentration of Mn, Fe, and Al.

Response	Source	^a^ DF	^b^ SS	^c^ MS	*F*-Value	Prob > F
Turbidity (NTU)	Regression	14	5420.79	387.2	739.70	<0.0001
	Residual	33	17.27	0.52		
	Lack-of-fit	21	3.45	0.64	2.01	0.11
	Pure error	12	3.82	0.32		
Mn (g/L)	Regression	14	0.01	9.39 × 10^−4^	1.18	0.33
	Residual	33	0.03	7.95 × 10^−4^		
	Lack-of-fit	21	6.73 × 10^−3^	3.20 × 10^−4^	0.2	0.99
	Pure error	12	0.02	1.63 × 10^−3^		
Fe (g/L)	Regression	14	4.74	0.34	14.12	<0.0001
	Residual	33	0.79	0.02		
	Lack-of-fit	21	0.62	0.03	2.06	0.10
	Pure error	12	0.17	0.01		
Al (g/L)	Regression	14	108.92	7.78	63.34	<0.0001
	Residual	33	4.05	0.12		
	Lack-of-fit	21	2.51	0.12	0.93	0.58
	Pure error	12	1.55	0.13		

^a^ DF: degree of freedom; ^b^ SS: sum of squares; ^c^ MS: mean of squares (=SS/DF).

**Table 5:** ANOVA analysis for quadratic model for each response.

Response	R^2^	Adjusted R^2^_adj_	Predicted R^2^	Adequate Precision
Turbidity	0.99	0.99	0.99	142.43
Mn	0.33	0.05	−0.01	3.53
Fe	0.86	0.80	0.68	16.92
Al	0.96	0.95	0.93	41.25

## Data Availability

Not applicable.

## Supplementary Materials

The following are available online at https://www.mdpi.com/1660-4601/18/17/9164/s1, Table S1: Central composite design (CCD) for experiment with actual and predicted responses of turbidity (NTU), residual concentration of Mn (mg/L), Fe (mg/L) and Al (mg/L); Table S2: Response surface models fitting correlating the responses (turbidity, concentration of Mn, Fe and Al), categorical (alum, PAC, ACH and chitosan) and numerical factors (A: coagulant dosage; B: initial pH) with their corresponding coefficient of the linear numerical factors, 2nd order numerical factors and two numerical interaction factors obtained by the regression analysis.
