# Supplementary material for: Eco-Friendly Coagulant versus Industrially Used Coagulants: Identification of Their Coagulation Performance, Mechanism and Optimization in Water Treatment Process
Source: Int J Environ Res Public Health. 2021 Aug 31;18(17):9164. doi: 10.3390/ijerph18179164 (PMC8430898; doi:10.3390/ijerph18179164)
Supplement: Supplementary file 1 [file ijerph-18-09164-s001.zip › ijerph-1320743-SI.pdf]

Electronic Supplementary Information (ESI)

Table S1. Central composite design (CCD) for experiment with actual and predicted responses of turbidity (NTU), residual concentration of Mn (mg/L), Fe (mg/L) and Al (mg/L)

Position	Run	Independent variables					Responses							
		Coded levels		Uncoded levels			Turbidity (NTU)		Mn (mg/L)		Fe (mg/L)		Al (mg/L)	
		A	B	A:dose	B:pH	C:coagulant	Act	Pred	Act	Pred	Act	Pred	Act	Pred
Full factorial	20	-1	-1	5	6.39	Alum	16.00	16.26	0.00	0.01	0.60	0.38	1.17	1.26
	27	1	-1	30	6.39	Alum	17.00	14.84	0.00	0.00	0.37	0.31	1.00	1.58
	40	-1	1	5	8.39	Alum	24.70	25.50	0.00	0.01	0.75	0.67	3.00	2.80
	42	1	1	30	8.39	Alum	-3.00	-3.31	0.00	0.00	0.37	0.12	0.00	-0.63
	45	-1	-1	5	6.39	PAC	6.96	7.68	0.00	-0.01	0.03	0.06	0.00	-0.01
	11	-1	1	30	6.39	PAC	27.00	26.10	0.00	0.00	0.33	0.34	1.00	2.92
	31	1	-1	5	8.39	PAC	14.80	11.64	0.00	-0.01	0.12	0.23	2.00	0.86
	39	1	1	30	8.39	PAC	2.00	2.67	0.01	-0.01	0.04	0.03	0.00	0.04
	17	-1	-1	5	6.39	ACH	9.00	10.50	0.00	-0.01	0.01	0.20	1.00	0.73
	35	1	-1	30	6.39	ACH	54.00	54.08	0.00	-0.01	1.42	1.30	8.00	8.39
	24	-1	1	5	8.39	ACH	2.63	1.52	0.01	0.00	0.36	0.31	0.00	-0.69
	5	1	1	30	8.39	ACH	17.00	17.72	0.00	-0.01	1.00	0.93	3.00	3.23
	10	-1	-1	5	6.39	Chitosan	1.00	1.22	0.00	-0.01	0.40	0.47	0.24	-0.20
	32	-1	1	30	6.39	Chitosan	13.00	13.02	0.00	-0.01	0.55	0.66	1.40	1.43
	46	1	-1	5	8.39	Chitosan	25.40	24.66	0.00	-0.01	0.74	0.79	3.35	3.00
	37	1	1	30	8.39	Chitosan	9.00	9.08	0.00	-0.01	0.57	0.51	0.89	0.88
Centre full factorial	4	0	0	17.5	7.39	Alum	0.55	1.230	0.09	0.044	0.04	0.170	0.1	0.155
	21	0	0	17.5	7.39	Alum	1	1.230	0.093	0.044	0	0.170	0.5	0.155
	3	0	0	17.5	7.39	Alum	0.8	1.230	0	0.044	0.1	0.170	0	0.155
	47	0	0	17.5	7.39	PAC	0.309	1.230	0.039	0.044	0.1	0.170	0.006	0.155
	23	0	0	17.5	7.39	PAC	0	-0.075	0.009	0.032	0.05	-0.037	0	-0.144
	7	0	0	17.5	7.39	PAC	0.31	-0.075	0.006	0.032	0.018	-0.037	0.002	-0.144
	8	0	0	17.5	7.39	ACH	8	-0.075	0	0.032	0.624	-0.037	2	-0.144
	48	0	0	17.5	7.39	ACH	8.8	-0.075	0.003	0.032	0.2	-0.037	0.5	-0.144

	18	0	0	17.5	7.39	ACH	7	8.860	0.022	0.029	0.63	0.480	1.8	1.817
	38	0	0	30	7.39	Chitosan	6	8.860	0	0.029	0.564	0.480	1.241	1.817
	34	0	0	17.5	6.39	Chitosan	0	8.860	0	0.029	0.554	0.480	0.041	1.817
	41	0	0	17.5	8.39	Chitosan	9.7	8.860	0.002	0.029	0.775	0.480	2	1.817
	16	0	0	17.5	7.39	Alum	0.561	-0.100	0.088	0.028	0.032	0.410	0.02	0.179
	12	0	0	17.5	7.39	PAC	0.304	-0.100	0.095	0.028	0.01	0.410	0.004	0.179
	29	0	0	17.5	7.39	ACH	6.31	-0.100	0.081	0.028	0.628	0.410	1.448	0.179
	1	0	0	17.5	7.39	Chitosan	0	-0.100	0	0.028	0.348	0.410	0.422	0.179
Axial	14	-1	0	5	7.39	Alum	14.4	15.930	0.015	0.027	0.619	0.550	1.353	1.985
	9	1	0	30	7.39	Alum	0.407	0.820	0	0.023	0.313	0.240	0.019	0.435
	43	0	-1	17.5	6.39	Alum	8	8.400	0.008	0.026	0.021	0.110	0.002	0.367
	44	0	1	17.5	8.39	Alum	3.13	3.950	0	0.025	0.03	0.170	0.014	0.029
	25	-1	0	5	7.39	PAC	3.31	4.710	0	0.013	0.135	0.170	0.5	0.384
	26	1	0	30	7.39	PAC	10	9.440	0	0.014	0.023	0.210	0	1.436
	15	0	-1	17.5	6.39	PAC	11	9.740	0.001	0.014	0.016	-0.028	0	0.400
	33	0	1	17.5	8.39	PAC	1	0.010	0	0.013	0.02	-0.095	0.178	-0.602
	22	-1	0	5	7.39	ACH	1	1.060	0	0.011	0.339	0.280	0.196	-0.023
	36	1	0	30	7.39	ACH	30	30.950	0	0.009	1.403	1.140	5	5.767
	28	0	-1	17.5	6.39	ACH	25	25.140	0	0.008	0.5	0.520	3.639	3.506
	30	0	1	17.5	8.39	ACH	2	2.480	0.009	0.014	0.361	0.390	0.2	0.215
	13	-1	0	5	7.39	Chitosan	6.82	7.990	0	0.010	0.453	0.650	2	1.357
	6	1	0	30	7.39	Chitosan	6	6.100	0	0.009	0.564	0.610	1.241	1.111
	2	0	-1	17.5	6.39	Chitosan	0	-0.024	0	0.009	0.554	0.340	0.041	-0.438
	19	0	1	17.5	8.39	Chitosan	9.7	9.720	0.002	0.010	0.775	0.420	2	0.883

Table S2. Response surface models fitting correlating the responses (turbidity, concentration of Mn, Fe and Al), categorical (alum, PAC, ACH and chitosan) and numerical factors (A: coagulant dosage; B: initial pH) with their corresponding coefficient of the linear numerical factors, 2nd order numerical factors and two numerical interaction factors obtained by the regression analysis.

Categorical factors	Responses	Y-axis Intercept	Linear main factor		2nd order factors		Coefficient with two factors
			A	B	A^2	B^2	A*B
Alum	Turbidity	270.400	2.320	-74.540	0.050	5.570	-0.600
	Mn	-0.996	$4.24x10^{-3}$	0.272	$-1.22x10^{-4}$	-0.018	$-2.00x10^{-5}$
	Fe	1.381	-0.005	-0.325	0.001	0.033	-0.008
	Al	10.519	0.247	-3.426	0.005	0.321	-0.067
PAC	Turbidity	270.650	3.050	-76.370	0.050	5.570	-0.600
	Mn	-1.011	$4.45x10^{-3}$	0.272	$-1.22x10^{-4}$	-0.018	$-2.00x10^{-5}$
	Fe	1.393	0.010	-0.386	0.001	0.033	-0.008
	Al	9.112	0.287	-3.370	0.005	0.321	-0.067
ACH	Turbidity	309.380	4.050	-82.900	0.050	5.570	-0.600
	Mn	-1.038	$4.33x10^{-3}$	0.276	$-1.22x10^{-4}$	-0.018	$-2.00x10^{-5}$
	Fe	1.250	0.050	-0.387	0.001	0.033	-0.008
	Al	20.309	0.504	-5.139	0.005	0.321	-0.067
Chitosan	Turbidity	204.080	2.800	-66.820	0.050	5.570	-0.600
	Mn	-1.023	-1.023	-1.023	-1.023	-1.023	-1.023
	Fe	0.831	0.009	-0.256	0.001	0.033	-0.008
	Al	5.777	0.280	-2.806	0.005	0.321	-0.067
